# Author Correction: Proteogenomic characterization of 2002 human cancers reveals pan-cancer molecular subtypes and associated pathways

**DOI:** 10.1038/s41467-022-32539-y

**Published:** 2022-08-10

**Authors:** Yiqun Zhang, Fengju Chen, Darshan S. Chandrashekar, Sooryanarayana Varambally, Chad J. Creighton

**Affiliations:** 1 Dan L. Duncan Comprehensive Cancer Center Division of Biostatistics, Baylor College of Medicine, Houston, TX USA; 2 Comprehensive Cancer Center, University of Alabama at Birmingham, Birmingham, AL 35233 USA; 3 Division of Molecular and Cellular Pathology, Department of Pathology, University of Alabama at Birmingham, Birmingham, AL 35233 USA; 4 The Informatics Institute, University of Alabama at Birmingham, Birmingham, AL 35233 USA; 5 Department of Bioinformatics and Computational Biology, The University of Texas MD Anderson Cancer Center, Houston, TX USA; 6 Human Genome Sequencing Center, Baylor College of Medicine, Houston, TX 77030 USA; 7 Department of Medicine, Baylor College of Medicine, Houston, TX USA

**Keywords:** Cancer genomics, Proteome informatics, Protein analysis, HIPPO signalling, Protein-protein interaction networks

Correction to: *Nature Communications* 10.1038/s41467-022-30342-3, published online 13 May 2022.

The original version of this Article contained an error in the ‘De novo proteome-based subtypes’ section of the Results, which incorrectly read ‘s3 and s11 cell lines had consistent negative correlations in contrast to the other subtypes (Fig. 4d). This observation indicated that s3 and s11 cell lines (and, by extension, their tumor counterparts) tended to express essential genes highly (Fig. 4e).’ The correct version states ‘s10’ in place of ‘s11’.

The legend of Figure 4 originally read, ‘c, Pearson correlation between differential protein expression patterns versus gene effect scores based on Cancer Dependency Map (DepMap) CRISPR assays (with low scores denoting essential genes), whereby s3 and s11 cell lines tend to have high expression of essential genes. e For s3 and s11 cell lines, associated patterns for the sets of genes having both high expression and low gene effect scores across cell lines (with at least half of cell lines having normalized expression >0.5 SD from the median and gene effect scores <−0.5).’ The correct version states ‘s10’ in place of ‘s11’.

These errors have now been corrected in both the PDF and HTML versions of the Article.

