# Supplementary material for: Proteogenomic characterization of 2002 human cancers reveals pan-cancer molecular subtypes and associated pathways
Source: Nat Commun. 2022 May 13;13:2669. doi: 10.1038/s41467-022-30342-3 (PMC9106650; doi:10.1038/s41467-022-30342-3)
Supplement: Supplementary file 1 — Supplementary Information [file 41467_2022_30342_MOESM1_ESM.pdf]

Supplementary Information

Zhang, Chen, Chandrashekar et al. “Proteogenomic characterization of 2002 human cancers reveals pan-cancer molecular subtypes and associated pathways”

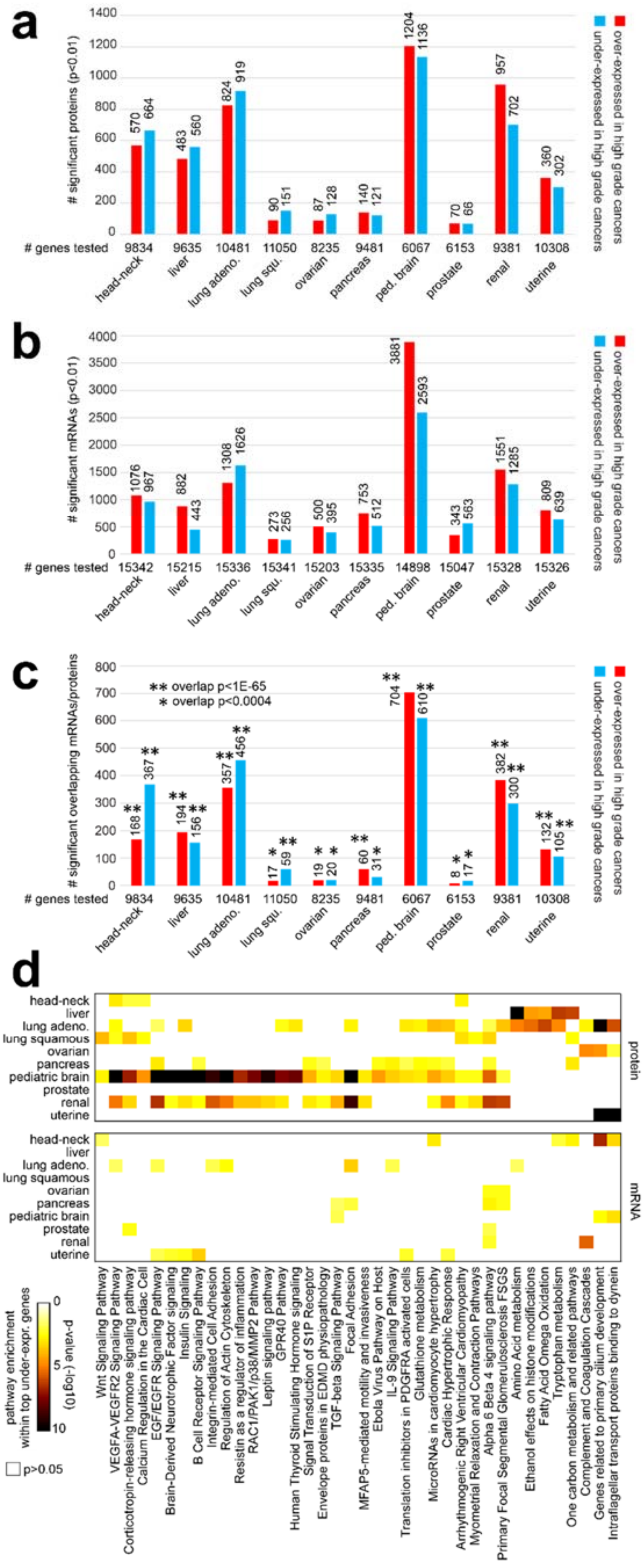
a
b
c
d
over-expressed in high grade cancers
under-expressed in high grade cancers
significant proteins (p<0.01)
significant mRNAs (p<0.01)
significant overlapping mRNAs/proteins
genes tested
head-neck
liver
lung adeno.
lung squ.
ovarian
pancreas
ped. brain
prostate
renal
uterine
** overlap p<1E-65
* overlap p<0.0004
lung squamous
pediatric brain
protein
mRNA
pathway enrichment within top under-expr. genes
p-value (-log10)
p>0.05
Wnt Signaling Pathway
VEGFA-VEGFR2 Signaling Pathway
Corticotropin-releasing hormone signaling pathway
Calcium Regulation in the Cardiac Cell
EGF/EGFR Signaling Pathway
Brain-Derived Neurotrophic Factor signaling
Insulin Signaling
B Cell Receptor Signaling Pathway
Integrin-mediated Cell Adhesion
Regulation of Actin Cytoskeleton
Resistin as a regulator of inflammation
RAC1/PAK1/p38/MMP2 Pathway
Leptin signaling pathway
GPR40 Pathway
Human Thyroid Stimulating Hormone signaling
Signal Transduction of S1P Receptor
Envelope proteins in EDMD physiopathology
TGF-beta Signaling Pathway
Focal Adhesion
MFAP5-mediated motility and invasiveness
Ebola Virus Pathway on Host
IL-9 Signaling Pathway
Translation inhibitors in PDGFRA activated cells
Glutathione metabolism
MicroRNAs in cardiomyocyte hypertrophy
Cardiac Hypertrophic Response
Arrhythmogenic Right Ventricular Cardiomyopathy
Myometrial Relaxation and Contraction Pathways
Alpha 6 Beta 4 signaling pathway
Primary Focal Segmental Glomerulosclerosis FSGS
Amino Acid metabolism
Ethanol effects on histone modifications
Fatty Acid Omega Oxidation
Tryptophan metabolism
One carbon metabolism and related pathways
Complement and Coagulation Cascades
Genes related to primary cilium development
Intraflagellar transport proteins binding to dynein

Supplementary Figure 1, related to Figure 2. Additional information on proteomic and transcriptomic signatures of high-grade cancers. (a) For each indicated cancer type, numbers of top differentially expressed proteins ($p < 0.01$, Pearson correlation using log-transformed data), associated with higher cancer grade. The numbers of unique genes tested are indicated for each comparison (involving proteins for which measurements were made in over half of samples profiled). **(b)** Similar to part a, but for differentially expressed mRNAs. The numbers of unique genes tested are indicated for each comparison. **(c)** Overlapping top protein and mRNA features ($p < 0.01$ for protein and $p < 0.01$ for mRNA, based on shared gene) by comparisons according to grade. P values for significance of overlap by one-sided Fisher's exact tests. **(d)** Significance of enrichment (by one-sided Fisher's exact test) for wikiPathway gene sets with the respective sets of proteins and mRNAs under-expressed ($p<0.01$, Pearson) with tumor grade for each cancer type represented. The set of pathways represented were significant within the under-expressed proteins for at least one cancer type with FDR<10% and for at least two cancer types with $p<0.01$.

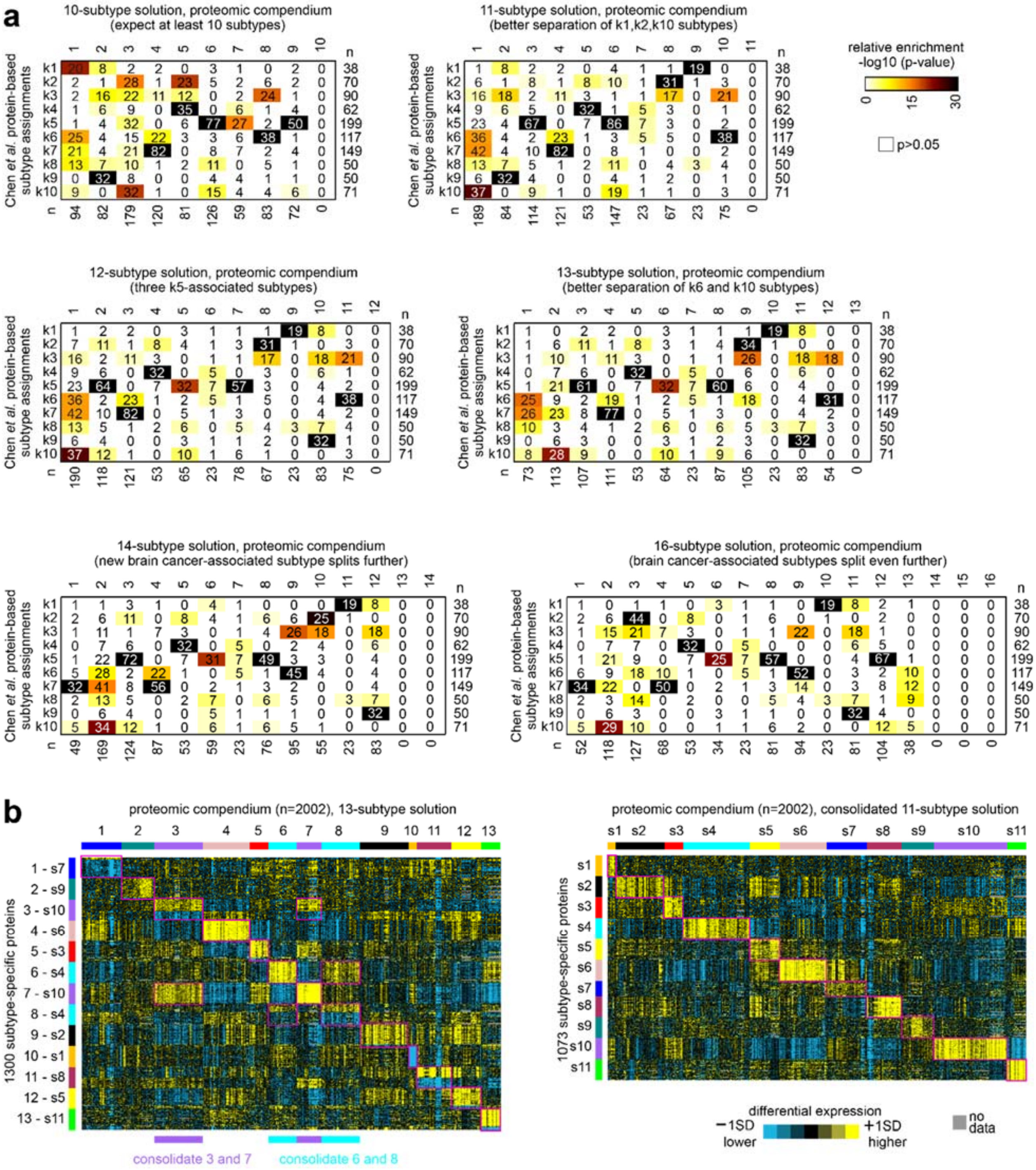
a
10-subtype solution, proteomic compendium
(expect at least 10 subtypes)
11-subtype solution, proteomic compendium
(better separation of k1,k2,k10 subtypes)
relative enrichment
-log10 (p-value)
0
15
30
p>0.05
12-subtype solution, proteomic compendium
(three k5-associated subtypes)
13-subtype solution, proteomic compendium
(better separation of k6 and k10 subtypes)
14-subtype solution, proteomic compendium
(new brain cancer-associated subtype splits further)
16-subtype solution, proteomic compendium
(brain cancer-associated subtypes split even further)
Chen et al. protein-based subtype assignments
n
b
proteomic compendium (n=2002), 13-subtype solution
proteomic compendium (n=2002), consolidated 11-subtype solution
1300 subtype-specific proteins
1 - s7
2 - s9
3 - s10
4 - s6
5 - s3
6 - s4
7 - s10
8 - s4
9 - s2
10 - s1
11 - s8
12 - s5
13 - s11
consolidate 3 and 7
consolidate 6 and 8
1073 subtype-specific proteins
s1
s2
s3
s4
s5
s6
s7
s8
s9
s10
s11
differential expression
−1SD
lower
+1SD
higher
no data

Supplementary Figure 2, related to Figure 3. Determining the number of *de novo* pan-cancer proteomic subtypes. (a) Consensus ward linkage hierarchical clustering identified k = 2 to k = 20 subtypes, based on proteomic data from 2002 human tumors (referred to here as the "proteomic compendium"). The significance of overlap of previously identified pan-cancer proteomic subtypes[1] (k1-k10, rows) with subtypes obtained from the current proteomic compendium dataset. Subtype solutions from 10 subtypes to 16 subtypes are considered here. P-values by one-sided Fisher's exact test. We expected at least ten subtypes, based on previous studies. With an 11-subtype solution, we observed better separation of the previously identified k1, k2, and k10 subtypes. With a 12-subtype solution, we observed three distinct k5-associated subtypes. With a 13-subtype solution, we observed better separation of k6 and k10 subtypes. With successive subtype solutions (14- and 16-subtype solutions represented here), the brain cancer-associated subtype ("s11" subtype in main Figure 3) splits further and further, but with no distinctive biology being associated with these subdivisions. The 13-subtype solution was therefore selected and explored further below. **(b)** Across 2002 tumor proteomic profiles, differential expression patterns (values normalized within each tissue-based cancer type; SD, standard deviation from the median) for a set of 1300 proteins (left) and for another set of 1073 proteins (right) found to best distinguish between the respective subtypes considered (top ~100 over-expressed proteins for each subtype). On the left, the 13-subtype solutions from consensus ward linkage hierarchical clustering (part a) is considered, where visually, subtypes 3 and 7 look very similar to each other, and subtypes 6 and 8 look similar to each other. Therefore, we both consolidated subtypes 3 and 7 into one subtype ("s10") and consolidated subtypes 6 and 8 into another subtype ("s4") to arrive at the final 11 subtype solution, represented on the right.

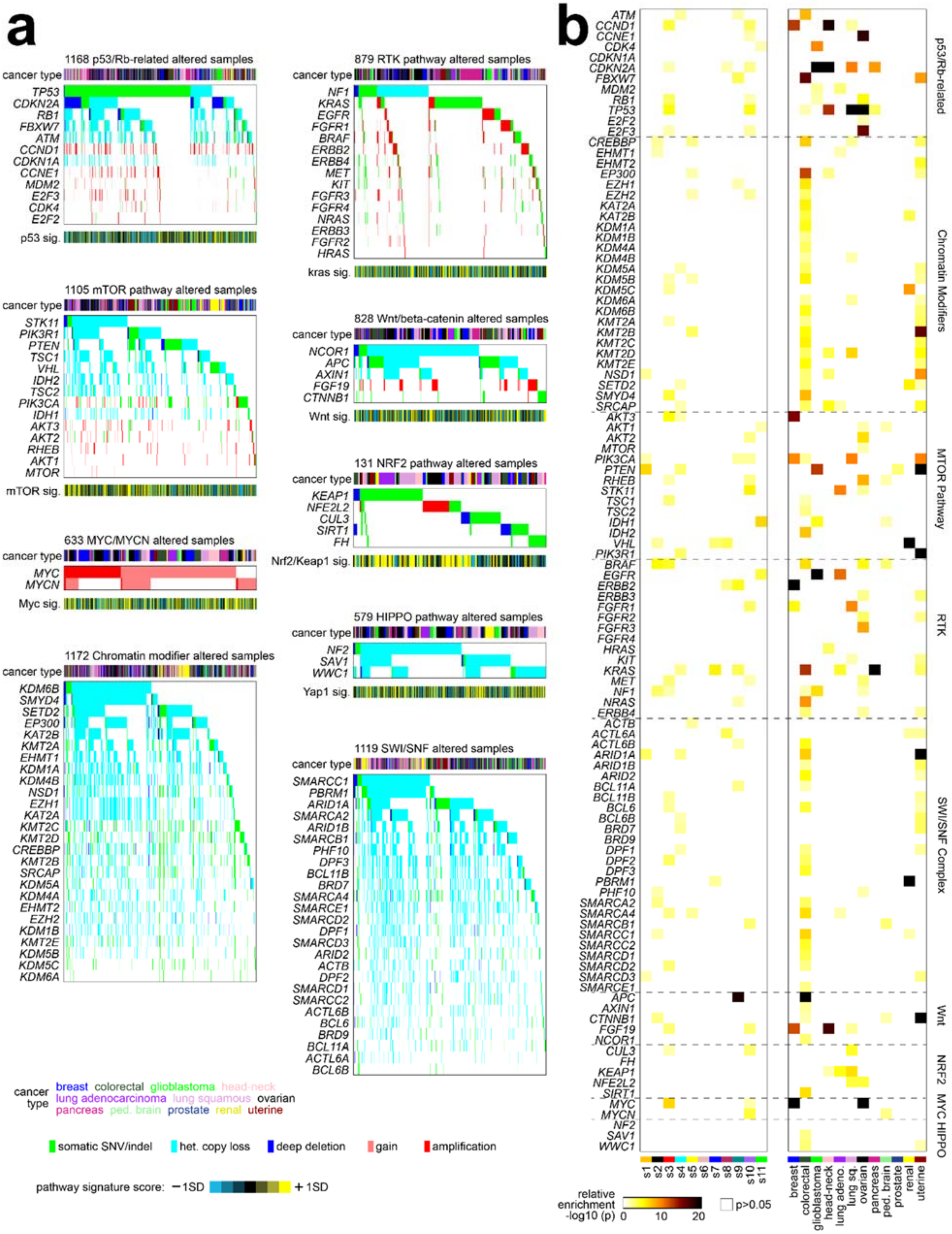
a
1168 p53/Rb-related altered samples
cancer type
TP53
CDKN2A
RB1
FBXW7
ATM
CCND1
CDKN1A
CCNE1
MDM2
E2F3
CDK4
E2F2
p53 sig.
879 RTK pathway altered samples
NF1
KRAS
EGFR
FGFR1
BRAF
ERBB2
ERBB4
MET
KIT
FGFR3
FGFR4
NRAS
ERBB3
FGFR2
HRAS
kras sig.
1105 mTOR pathway altered samples
STK11
PIK3R1
PTEN
TSC1
VHL
IDH2
TSC2
PIK3CA
IDH1
AKT3
AKT2
RHEB
AKT1
MTOR
mTOR sig.
828 Wnt/beta-catenin altered samples
NCOR1
APC
AXIN1
FGF19
CTNNB1
Wnt sig.
131 NRF2 pathway altered samples
KEAP1
NFE2L2
CUL3
SIRT1
FH
Nrf2/Keap1 sig.
633 MYC/MYCN altered samples
MYC
MYCN
Myc sig.
579 HIPPO pathway altered samples
NF2
SAV1
WWC1
Yap1 sig.
1172 Chromatin modifier altered samples
KDM6B
SMYD4
SETD2
EP300
KAT2B
KMT2A
EHMT1
KDM1A
KDM4B
NSD1
EZH1
KAT2A
KMT2C
KMT2D
CREBBP
KMT2B
SRCAP
KDM5A
KDM4A
EHMT2
EZH2
KDM1B
KMT2E
KDM5B
KDM5C
KDM6A
1119 SWI/SNF altered samples
SMARCC1
PBRM1
ARID1A
SMARCA2
ARID1B
SMARCB1
PHF10
DPF3
BCL11B
BRD7
SMARCA4
SMARCE1
SMARCD2
DPF1
SMARCD3
ARID2
ACTB
DPF2
SMARCD1
SMARCC2
ACTL6B
BCL6
BRD9
BCL11A
ACTL6A
BCL6B
cancer type
breast colorectal glioblastoma head-neck
lung adenocarcinoma lung squamous ovarian
pancreas ped. brain prostate renal uterine
somatic SNV/indel
het. copy loss
deep deletion
gain
amplification
pathway signature score: − 1SD + 1SD
b
p53/Rb-related
Chromatin Modifiers
MTOR Pathway
RTK
SWI/SNF Complex
Wnt
NRF2
MYC
HIPPO
s1 s2 s3 s4 s5 s6 s7 s8 s9 s10 s11
breast colorectal glioblastoma head-neck lung adeno. lung sq. ovarian pancreas ped. brain prostate renal uterine
relative enrichment -log10 (p)
0 10 20
p>0.05

Supplementary Figure 3, related to Figure 7. Somatic alteration patterns across cancers by pathway annotation. (a) For the pathways from main Figure 7, somatic alteration events involving each gene included in the pathway are represented. Where applicable, the corresponding pathway signature patterns are shown (for p53 signature, based on mRNA; for the other signatures, based on protein). **(b)** For each cancer type and pan-cancer proteomic subtype, significance of enrichment (one-sided Fisher's exact test) of mutation events for each gene within the given proteomic subtype/cancer type versus the rest of the tumors.

<u>References</u>

1. Chen, F., Chandrashekar, D., Varambally, S. & Creighton, C. Pan-cancer molecular subtypes revealed by mass-spectrometry-based proteomic characterization of more than 500 human cancers. *Nat Commun* **10**, 5679 (2019).
