# Supplementary material for: Proteogenomic characterization of 2002 human cancers reveals pan-cancer molecular subtypes and associated pathways
Source: Nat Commun. 2022 May 13;13:2669. doi: 10.1038/s41467-022-30342-3 (PMC9106650; doi:10.1038/s41467-022-30342-3)
Supplement: Supplementary file 3 — Description of Additional Supplementary Files [file 41467_2022_30342_MOESM3_ESM.pdf]

Description of Additional Supplementary Files

Supplementary Data 1. Tumor-level sample annotation table. Provided as an Excel file. Tumor-level table on the 2002 human tumors in the proteomic compendium dataset includes the following types of information: molecular profiling data platforms available for the tumor sample, proteome-based subtype membership, tumor grade, pathway-level genetic and genomic alterations, protein and mRNA signatures, and normalized levels of selected proteins and phospho-proteins.

Supplementary Data 2. Protein-level correlations. Provided as an Excel file. For 15439 total proteins represented in the proteomic compendium dataset, correlations with mRNA, correlations with CNA, correlations with tumor grade, correlations with overall CNA burden, and correlations with *TP53* or *KRAS* mutant status are provided. Also provided for each protein are antibody availability information from The Human Protein Atlas (proteinatlas.org). The Human Protein Atlas includes antibody validation information for immunohistochemistry and immunocytochemistry/IF. For more information on a given antibody, one can either do a web search on the antibody accession number or search for the gene at www.proteinatlas.org then select the 'Antibody Information' option. Additional information on antibody reliability information is available at https://www.proteinatlas.org/about/antibody+validation.

Supplementary Data 3. mRNA-level correlations. Provided as an Excel file. For 15439 mRNA transcripts for which total proteins are represented in the proteomic compendium dataset, correlations with CNA, correlations with tumor grade, correlations with overall CNA burden, and correlations with *TP53* or *KRAS* mutant status are provided.

Supplementary Data 4. Protein-level subtype associations. Provided as an Excel file. Results based on the proteomic compendium dataset. For each protein, correlations with each of the proteome-based pan-cancer subtypes (s1-s11) are provided. The top 100 subtype-specific proteins high for each subtype (top 73 for s7) are indicated. Also provided for each protein are any association in the DrugBank database. The subset of 225 proteins that both had a DrugBank association and were in the set of 1073 proteins that best distinguish between the ten proteome-based subtypes (Figure 3b) and provided in a separate Excel tab. The sets of protein features used to classify the external proteomic datasets (TCGA-RPPA, and CCLE-mass spectrometry) are also provided. Calculations to assign subtype based on the classifier are provided in Excel. For the TCGA-RPPA protein features, RPPA antibody information (company, catalog number, species, validation status) is provided where available.

Supplementary Data 5. GO and pathway associations. Provided as an Excel file. For 15439 total proteins represented in the proteomic compendium dataset, membership is selected gene sets of interest in the study, e.g., based on Gene Ontology or wikiPathways annotation, are indicated.

Supplementary Data 6. Phospho-protein-level correlates. Provided as an Excel file. For 5419 phospho-proteins with data for >50% of samples in at least seven cancer types, correlations with total protein and associations with proteome-based subtypes s1-s11 are provided.
