# Supplementary material for: Proteogenomic characterization of 2002 human cancers reveals pan-cancer molecular subtypes and associated pathways
Source: Nat Commun. 2022 May 13;13:2669. doi: 10.1038/s41467-022-30342-3 (PMC9106650; doi:10.1038/s41467-022-30342-3)
Supplement: Supplementary file 10 — Reporting Summary [file 41467_2022_30342_MOESM10_ESM.pdf]

nature research

Corresponding author(s): Chad Creighton

Last updated by author(s): Mar 23, 2022

Reporting Summary

Nature Research wishes to improve the reproducibility of the work that we publish. This form provides structure for consistency and transparency in reporting. For further information on Nature Research policies, see Authors & Referees and the Editorial Policy Checklist.

Statistics

For all statistical analyses, confirm that the following items are present in the figure legend, table legend, main text, or Methods section.

n/a	Confirmed	
☐	☒	The exact sample size (*n*) for each experimental group/condition, given as a discrete number and unit of measurement
☐	☒	A statement on whether measurements were taken from distinct samples or whether the same sample was measured repeatedly
☐	☒	The statistical test(s) used AND whether they are one- or two-sided<br>*Only common tests should be described solely by name; describe more complex techniques in the Methods section.*
☐	☒	A description of all covariates tested
☐	☒	A description of any assumptions or corrections, such as tests of normality and adjustment for multiple comparisons
☐	☒	A full description of the statistical parameters including central tendency (e.g. means) or other basic estimates (e.g. regression coefficient) AND variation (e.g. standard deviation) or associated estimates of uncertainty (e.g. confidence intervals)
☐	☒	For null hypothesis testing, the test statistic (e.g. *F*, *t*, *r*) with confidence intervals, effect sizes, degrees of freedom and *P* value noted<br>*Give P values as exact values whenever suitable.*
☐	☒	For Bayesian analysis, information on the choice of priors and Markov chain Monte Carlo settings
☐	☒	For hierarchical and complex designs, identification of the appropriate level for tests and full reporting of outcomes
☐	☒	Estimates of effect sizes (e.g. Cohen's *d*, Pearson's *r*), indicating how they were calculated

Our web collection on statistics for biologists contains articles on many of the points above.

Software and code

Policy information about availability of computer code

Data collection	All data used in this study are publicly available. CPTAC data are available through the CPTAC data portal (https://cptac-data-portal.georgetown.edu/cptacPublic/). The compendium datasets of molecular profiles for total protein, phospho-protein, and mRNA—compiled as part of our study—are available through GitHub [https://github.com/chadcreighton/cancer-proteomics-compendium-n2002]. Each molecular dataset is uploaded on GitHub as a series of separate project files, using a common protein feature set with the same ordering across files. One can concatenate the files together. Each molecular dataset has a common sample set, allowing one to derive correlations between datasets (e.g., between mRNA and protein). The phospho-protein datasets consist of 5419 phospho-protein features that had available data for >50% of samples in at least seven cancer types. No software was used for data collection. Microsoft Excel was used to transform expression values and to concatenate datasets as described in Methods.
Data analysis	No specialized code was written for this study. Source code in R (written using version 4.1.1) for identifying de novo pan-cancer proteomic subtypes, using ConsensusClusterPlus method (version 1.59.0), with an example data table of proteomic expression for the top ~2000 variable genes across 2002 tumor, is available at Github [https://github.com/chadcreighton/cancer-proteomics-compendium-n2002]. Visualization using heat maps was performed using both JavaTreeview (version 1.1.6r4) and matrix2png (version 1.2.1). SigTerms is available at Sourceforge [http://sigterms.sourceforge.net/]

For manuscripts utilizing custom algorithms or software that are central to the research but not yet described in published literature, software must be made available to editors/reviewers. We strongly encourage code deposition in a community repository (e.g. GitHub). See the Nature Research guidelines for submitting code & software for further information.

Data

Policy information about availability of data

All manuscripts must include a data availability statement. This statement should provide the following information, where applicable:

- Accession codes, unique identifiers, or web links for publicly available datasets
- A list of figures that have associated raw data
- A description of any restrictions on data availability

All data used in this study are publicly available. Proteomics data are available for CPTAC and ICPC studies at the Proteomic Data Commons [https://pdc.cancer.gov/]. For CPTAC studies, transcriptome data, copy number data, and small somatic mutation data are available at the Genomic Data Commons [https://gdc.cancer.gov/]. TCGA data RNA-seq data are also available from the Broad Institute's Firehose data portal [https://gdac.broadinstitute.org]. The TCGA RPPA dataset is available from the TCPA portal [http://tcpaportal.org/tcpa/]. Cancer Cell Line Encyclopedia (CCLE) datasets are available from the CCLE website [http://www.broadinstitute.org/ccle]. For other published studies, molecular data availability information is provided in the associated publication. The compendium datasets of molecular profiles for total protein, phospho-protein, and mRNA—compiled as part of our study—are available through GitHub [https://github.com/chadcreighton/cancer-proteomics-compendium-n2002]. Each molecular dataset is uploaded on GitHub as a series of separate project files, using a common protein feature set with the same ordering across files. One can concatenate the files together. Each molecular dataset has a common sample set, allowing one to derive correlations between datasets (e.g., between mRNA and protein). The phospho-protein datasets consist of 5419 phospho-protein features that had available data for >50% of samples in at least seven cancer types. Any remaining data are available within the Article, Supplementary Information, or from the authors. Source data are provided with this paper.

Field-specific reporting

Please select the one below that is the best fit for your research. If you are not sure, read the appropriate sections before making your selection.

☒ Life sciences ☐ Behavioural & social sciences ☐ Ecological, evolutionary & environmental sciences

For a reference copy of the document with all sections, see nature.com/documents/nr-reporting-summary-flat.pdf

Life sciences study design

All studies must disclose on these points even when the disclosure is negative.

Sample size	We assembled a compendium dataset of mass spectrometry-based proteomics data of primary tumors from 14 cancer types and 17 individual studies (Data File S1). Most of these previous studies were led by either CPTAC or the International Cancer Proteogenome Consortium (ICPC). The cancer types represented in the proteomics compendium dataset were the following: Breast Invasive Carcinoma (n=230 tumors with proteomics data), Colorectal Adenocarcinoma (n=187), Gastric Cancer (n=80), Glioblastoma (n=100), Head and Neck Squamous Cell Carcinoma (n=108), Hepatocellular Carcinoma (n=165), Lung Adenocarcinoma (n=111), Lung Squamous Cell Carcinoma (n=110), Ovarian Serous Cystadenocarcinoma (n=269), Pancreatic Ductal Adenocarcinoma (n=137), Pediatric Brain Tumors (n=219), Prostate Adenocarcinoma (n=76), Renal Cell Carcinoma (n=110), and Uterine Corpus Endometrial Carcinoma (n=100). The 2002 tumors in the compendium represented 1982 patients, with the pediatric brain tumor dataset involving 219 tumors from 199 patients. The above studies analyzed the tumors using liquid chromatography-tandem mass spectrometry (LC-MS/MS) global proteomic and phosphoproteomic profiling. Of the 2002 human tumors with proteomics data, 1899 had corresponding RNA-seq data. Of the 2002 human tumors with proteomics data, 1837 had corresponding gene-level CNA data. Of the 2002 human tumors with proteomics data, 1698 had corresponding small somatic mutation data (SNVs and indels) by whole-exome or whole-genome sequencing. All proteogenomics studies with available multi-omics data to our knowledge were incorporated into the present study. Our study took advantage of the large number of tumors represented in our proteomic compendium dataset, whereby we could carry out tumor subset analyses involving sparse mutation events.
Data exclusions	No data were excluded from the analysis.
Replication	Not applicable, no experimental data. We did not run controlled experiments involving limited numbers of cell cultures or of mice.
Randomization	Not applicable, no experimental data. We did not carry out a controlled experimental data whereby subjects or mice were assigned to different experimental groups.
Blinding	Blinding not relevant, as samples were not allocated to experimental groups.

Reporting for specific materials, systems and methods

We require information from authors about some types of materials, experimental systems and methods used in many studies. Here, indicate whether each material, system or method listed is relevant to your study. If you are not sure if a list item applies to your research, read the appropriate section before selecting a response.

Materials & experimental systems

n/a	Involved in the study
☒	☐ Antibodies
☒	☐ Eukaryotic cell lines
☒	☐ Palaeontology
☒	☐ Animals and other organisms
☒	☐ Human research participants
☒	☐ Clinical data

Methods

n/a	Involved in the study
☒	☐ ChIP-seq
☒	☐ Flow cytometry
☒	☐ MRI-based neuroimaging
